# Prevalence and antimicrobial susceptibility profile of *Staphylococcus aureus* from meat, abattoir workers, equipment and water samples at Abergelle International Livestock Development PLC, Mekelle, Northern Ethiopia

**DOI:** 10.1186/s12866-026-04709-1

**Published:** 2026-01-15

**Authors:** Haftom Yirga Tsegay, Muuz Gebru Sahle, Biruk Mekonnen Woldie, Kedir Seid Abdelkadir

**Affiliations:** 1 Department of Veterinary Basics and Diagnostic Sciences, College of Veterinary Sciences, Mekelle University, Mekelle, Ethiopia; 2 Doctoral School of Biology and Environmental Sciences, University of Debrecen, Debrecen, Hungary

**Keywords:** Abattoir, Antimicrobial susceptibility, Isolation, Mekelle, Prevalence, *Staphylococcus aureus*

## Abstract

**Background:**

Raw meat is one of the main sources of food borne infections worldwide. *Staphylococcus aureus* (*S. aureus*) is one of the key organisms that is prevalent in contaminated meat, having different patterns of antimicrobial susceptibility to different antibiotics. This study aimed to determine the prevalence and antimicrobial susceptibility pattern of *S. aureus* in meat, abattoir workers, water, and equipment samples.

**Methods:**

A cross-sectional study was conducted from January to September 2023 at the Abergelle International Export Abattoir, Tigray, Ethiopia. Meat and equipment samples were collected via simple random sampling, while swabs from slaughter lines, personnel, and water were obtained by convenience sampling, all aseptically on a weekly basis after flaying process of slaughtering. A total of 400 samples were collected: 233 meat, 95 equipment, 24 slaughter line, 48 personnel, and 24 water samples. Antimicrobial susceptibility test was conducted on 20 randomly selected *S. aureus* isolates using the disk diffusion method. Data were entered in EpiData manager 4.6 and analyzed with SPSS 27 for descriptive and inferential statistics.

**Results:**

The overall prevalence of *S. aureus* was 29.3% (117/400). Isolation rates varied by sample type (*p* < 0.05): meat 18.5% (43/233), personnel 54.1% (26/48), equipment 37.8% (31/82), and water 12.5% (3/24), with knife swabs showing the highest contamination (83.3%, 20/24). Prevalence was higher in cattle slaughterhouse (33.7%, 85/252; AOR: 1.74; 95% CI: 1.07–2.82; *p* < 0.05) than shoat slaughterhouse (21.6%, 32/148) and higher during non-fasting periods (35.2%, 70/199; AOR = 1.69; 95% CI: 1.06–2.70; *p* < 0.05) than fasting periods (23.4%, 47/201). All isolates were fully susceptible to ciprofloxacin, chloramphenicol, and sulfamethoxazole-trimethoprim. Resistance was highest to penicillin-G (90%). Multidrug resistance (MDR) occurred in 20% of the isolates.

**Conclusion:**

The abattoir exhibited notable contamination with *S. aureus*, including multidrug-resistant strains, with particularly high contamination associated with processing equipment and personnel hands. These findings reveal critical hygiene gaps along the slaughter line and emphasize the need for targeted sanitation measures and routine antimicrobial susceptibility monitoring within a One Health framework to limit the spread of resistant organisms.

**Supplementary Information:**

The online version contains supplementary material available at 10.1186/s12866-026-04709-1.

## Background

Foodborne illnesses are widespread in many African countries, largely due to poor handling practices and limited food safety regulations [1–3]. Animal-source foods such as meat, milk, and eggs commonly carry *Staphylococcus aureus* (*S. aureus*), contributing to frequent foodborne outbreaks [4]. Meat is especially vulnerable to contamination at multiple stages, including slaughter, processing, storage, and distribution [5].

Meat can become contaminated through infected animals, poor hygiene, inadequate sterilization, or contaminated equipment and facilities, creating both economic losses and health risks due to *S. aureus* enterotoxins [6]. The nutrient-rich, near-neutral pH of meat and milk further supports bacterial growth [7]. *S. aureus*, a common colonizer of human and animal skin and nasal mucosa, is carried by roughly 30% of healthy individuals and is a major potential contamination source in abattoirs [8, 9].

*S. aureus* carries numerous virulence factors that make it a significant food safety and public health threat. Its enterotoxins, over 20 heat-stable, protease-resistant toxins, can cause classical food poisoning even after the bacteria are no longer viable [10]. These superantigens are frequently linked to outbreaks from contaminated meat, milk, and other animal products [11]. Genomic studies also show that enterotoxin expression is tightly regulated by prophages and global regulatory systems, adding to the organism’s pathogenicity [12–14]. Clinically, *S. aureus* can cause pneumonia, bacteremia, osteomyelitis, dermatitis, meningitis, endocarditis, and toxic shock syndrome [15–17], while in animals it commonly results in mastitis [18, 19], skin and soft tissue infections [20], and bumble foot in poultry [16].

Antimicrobial resistance (AMR) in *S. aureus* has emerged as a major One Health concern, driven by inappropriate antimicrobial use in livestock, weak regulatory systems, and insufficient food-safety controls [21]. Resistant strains, including methicillin-resistant *S. aureus* (MRSA) and multidrug-resistant *S. aureus*, are increasingly reported in food animals, animal products, and throughout the food chain [22]. In many low and middle-income regions, particularly sub-Saharan Africa, limited stewardship, over-the-counter access to antibiotics, and weak surveillance systems have accelerated the spread of resistant strains from animals to humans through direct contact, food handling, or consumption of contaminated products [23]. Similar trends are documented in East Africa, where rising resistance to tetracyclines, penicillins, and macrolides in livestock-associated *S. aureus* is a growing public health and food safety concern [24–26].

In Ethiopia, the irrational use and misuse of antimicrobials by health care providers, unskilled practitioners, and drug consumers are frequent and play a large role in changing the resistance level [27, 28]. Additionally, cultural practices involving the consumption of raw or undercooked meat may further heighten the risk of exposure as they are common in Ethiopian context [29, 30].

Although the Abergelle International Export Abattoir primarily targeted export markets, it also supplies local high-standard hotels and universities, making any contamination a potential public health concern. Despite these concerns, data on the prevalence and antimicrobial susceptibility of *S. aureus* in abattoir environments in Tigray, Ethiopia remains scarce, as most studies focus on clinical settings. This study addresses that gap by determining the prevalence and evaluating the antimicrobial susceptibility patterns of *S. aureus* in meat, personnel, water, and equipment at the Abergelle abattoir, providing evidence that can support both local and export-oriented meat safety interventions.

## Methods

### Study area

**Fig. 1:**
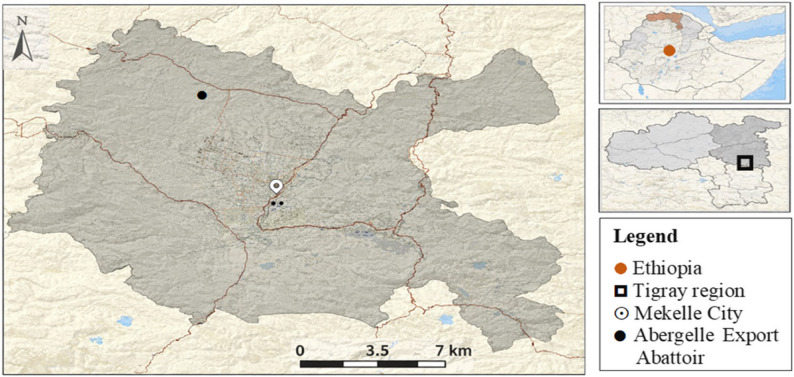
Map of Mekelle city (Arc GIS, 2025)

This study was conducted at the Abergelle International Export Abattoir, the only legally established large-scale export abattoir in the Tigray region of Ethiopia that meets recognized export standards. The facility is located 9 km north of Mekelle city and operates under the Abergelle International Livestock Development PLC established in 2005. It has modern slaughtering and processing infrastructure, including equipment imported from Germany. It has 18 cold rooms with a capacity of over 240 cattle and 960 sheep/goats per day. It uses municipal water supply serving all processing and sanitation activities. Although the abattoir has historically exported beef, mutton, goat meat, and offal to North Africa, the Middle East, and Asia, operations have recently shifted toward supplying local institutions due to market and technical challenges [31] (Fig1).

### Study design

A cross-sectional study design was employed to assess the prevalence and antimicrobial susceptibility patterns of *Staphylococcus aureus* (*S. aureus*) in an abattoir setting.

### Study period

The study was conducted from January to September 2023.

### Study population

The study population included slaughtered animals (bovine, caprine, and ovine), abattoir personnel, equipment, and the water used during slaughtering and processing. All slaughtered animals were male, due to their higher carcass yield and better dressing percentage, which is in line with export-oriented purchasing practices. Although sex-based differences in *S. aureus* colonization have not been consistently demonstrated in food animals, sampling only males may slightly limit generalizability. The animals were originated from farmer cooperatives and traders operating under intensive and semi-intensive systems in the Tigray, Amhara, and Afar regions of Ethiopia. Abattoir personnel participating in the study were workers involved in slaughtering, processing, packaging, and transporting beef, mutton, and goat meat.

### Sample size determination and sampling methodology

The sample size was determined using the standard formula for estimating proportions described by Thrusfield [32], given below. In the absence of prior prevalence data from the study area, an expected prevalence (P) of 50% was assumed, as this provides the maximum sample size and ensures adequate statistical power. Using a 95% confidence level (Z = 1.96) and a 5% margin of error (d), the minimum required sample size was 384. To increase precision, a total of 400 samples were collected.

$$\:n={\left(Z\right)}^{2}\:P\frac{1-P}{{d}^{2}}$$

The sampling contained 233 meat swabs (159 beef, 50 goat meat and 24 mutton), 95 equipment swabs (24 knife, 24 apron, 23 splitting axe and 24 slaughter line), 24 slaughter line swabs, 48 abattoir personnel swabs (24 nasal swabs and 24 hand swabs), and 24 water samples.

Sample allocation among species of animals was performed proportionally to the abattoir’s throughput during the study period. Cattle represent the predominant species processed at the facility; therefore, 159 meat swabs were collected from cattle, compared with 50 from goats and 24 from sheep. A simple random sampling technique was used to collect meat and equipment samples (a subset of the total per each collection day), while a convenience sampling technique was used to collect samples from the slaughter lines, water samples and abattoir personnel participating in slaughtering, evisceration, processing, and packaging of meat.

### Sample collection and transport

Samples were collected aseptically on a weekly basis (20 samples per week) after the flaying and evisceration process and abattoir personnel samples were collected during their active shifts to capture potential contamination. Equipment and water samples were collected before the beginning of the active processing, prior to any slaughter activities. To prevent cross-contamination, sterile materials were used throughout, gloves were changed between samples, and a new sterile swab or bottle was used for each sampling point. The species, source, and time of collection (fasting vs. non-fasting periods) were recorded for each sample. Observations of the general abattoir environment, lairages, slaughtering process, and hygiene measures were conducted using a checklist.

Sterile cotton-tipped swabs were used to sample carcasses, equipment, and personnel. Carcass swabs were taken from rump, flank, brisket, and neck over an approximately 100 cm² area. Entire surfaces of knives and splitting axes were swabbed, while apron and slaughter line surfaces were swabbed approximately 100 cm². For personnel sampling, nasal swabs were collected by gently inserting a sterile swab approximately 1–2 cm into each nostril and rotating it against the nasal mucosa. Hand swabs were collected by swabbing the entire surface of both hands, including palms, fingers, and interdigital spaces, using moistened sterile swabs. All swab tips were immediately placed into 10 mL of buffered peptone water after breaking off the shafts inside the test tubes to prevent contamination. For water samples, 1 mL of potable water which was used for washing carcasses and equipment was added to 9 mL of buffered peptone water. Samples were transported in ice boxes to the Microbiology Laboratory of the College of Veterinary Sciences, Mekelle University, and stored at + 4 °C until culturing. Sample collection and transportation was conducted according to standard procedures described by the International Standards Organization [33, 34].

### Isolation and identification

Identification of *S. aureus* was based on a combination of phenotypic and biochemical assays. Swab samples were first placed into 10 mL of buffered peptone water and incubated at 37 °C for 24 h for enrichment. After incubation, a loopful of the enriched culture was streaked onto 7% sheep blood agar and incubated aerobically at 37 °C for 24 h. Presumptive staphylococcal colonies, round, smooth, white to yellow, and β-haemolytic, were selected and sub-cultured on nutrient agar to obtain pure cultures. Representative colonies were subjected to Gram staining, and those showing Gram-positive cocci in clusters were retained for further testing. As a next step, catalase testing was performed by emulsifying a small amount of culture in 3% hydrogen peroxide; immediate bubble formation indicated a positive reaction. Each isolate was then streaked onto Mannitol Salt Agar (MSA) and incubated at 37 °C for 24 h; yellowing of the medium indicated mannitol fermentation. DNase activity was assessed by streaking isolates on DNase agar, incubating at 37 °C for 24 h, and flooding with HCl; a clear halo indicated DNase production. Tube coagulase test was performed by inoculating 0.5 mL of rabbit plasma with a loopful of culture and incubating at 37 °C, with readings at 1 h and 24 h; clot formation confirmed coagulase positivity.

Isolates positive for Gram-positive clustered cocci, catalase, mannitol fermentation, DNase, and coagulase were confirmed as *S. aureus*, while coagulase-negative isolates were excluded from *S. aureus* counts. *S. aureus* ATCC 25,923 was used as a quality control strain for culture and biochemical testing, and ambiguous results were resolved by repeat testing and comparison with the control strain.

### Antimicrobial susceptibility test (AST)

Antimicrobial sensitivity tests of the isolates were performed via the disk diffusion method [35] according to Clinical Laboratory Standards Institute (CLSI) M100, 30th edition [36].

Nine different antimicrobial discs for nine class of antimicrobials that commonly used for treating different bacterial diseases and considered important for human health were used for the test. The nine antimicrobials were: gentamycin (GEN-10 µg), chloramphenicol (C-30 µg), penicillin-G (P-10 units), erythromycin (E-15 µg), doxycycline (DO-30 µg), ciprofloxacin (CIP-5 µg), sulfamethoxazole-trimethoprim (COT-25 µg), cefoxitin (CFX-30 µg) and vancomycin (VA) were tested for twenty randomly selected isolates of *S. aureus. S. aureus* ATCC 25,923 was used as the quality control strain for the eight antimicrobials using the disk diffusion method. The minimum inhibitory concentration (MIC) through the broth microdilution method was applied for vancomycin sensitivity. *S. aureus* ATCC 29,213 was used as a quality control strain for vancomycin MIC.

The isolates were classified as resistant, intermediate or sensitive according to the Clinical Laboratory Standards Institute (CLSI) [36]. The interpretive criteria used for antimicrobial susceptibility testing of *S. aureus* is provided in Supplementary Table 1. Multidrug resistance (MDR) was determined according to the international standard proposed by Magiorakos et al.., as non-susceptibility to at least one agent in three or more different antimicrobial classes [37].

### Data management and analysis

Data were entered into EpiData manager 4.6 and analyzed using SPSS version 27. Descriptive statistics were used to summarize the data and calculate the prevalence of *S. aureus*. Univariable analyses were conducted using chi-square (χ²) tests and univariable logistic regression to estimate crude odds ratios (CORs) with 95% confidence intervals. Variables with a p-value ≤ 0.25 in univariable analysis were considered for inclusion in the multivariable logistic regression model. Multivariable logistic regression was performed to identify independent predictors of *S. aureus* contamination, and adjusted odds ratios (AORs) with 95% confidence intervals were calculated. Statistical significance was set at *p* < 0.05. Slaughter line samples, shoat slaughterhouse, fasting period, and sheep were used as reference categories. Multicollinearity was assessed using variance inflation factors (VIF), and model fit was evaluated using the Hosmer-Lemeshow goodness-of-fit test. The predictive performance of the final model was evaluated using the receiver operating characteristic (ROC) curve. Antimicrobial susceptibility results were summarized as percentages of resistant, intermediate, and susceptible isolates.

## Results

### Isolation rate of *Staphylococcus aureus* from the abattoir

**Fig. 2:**
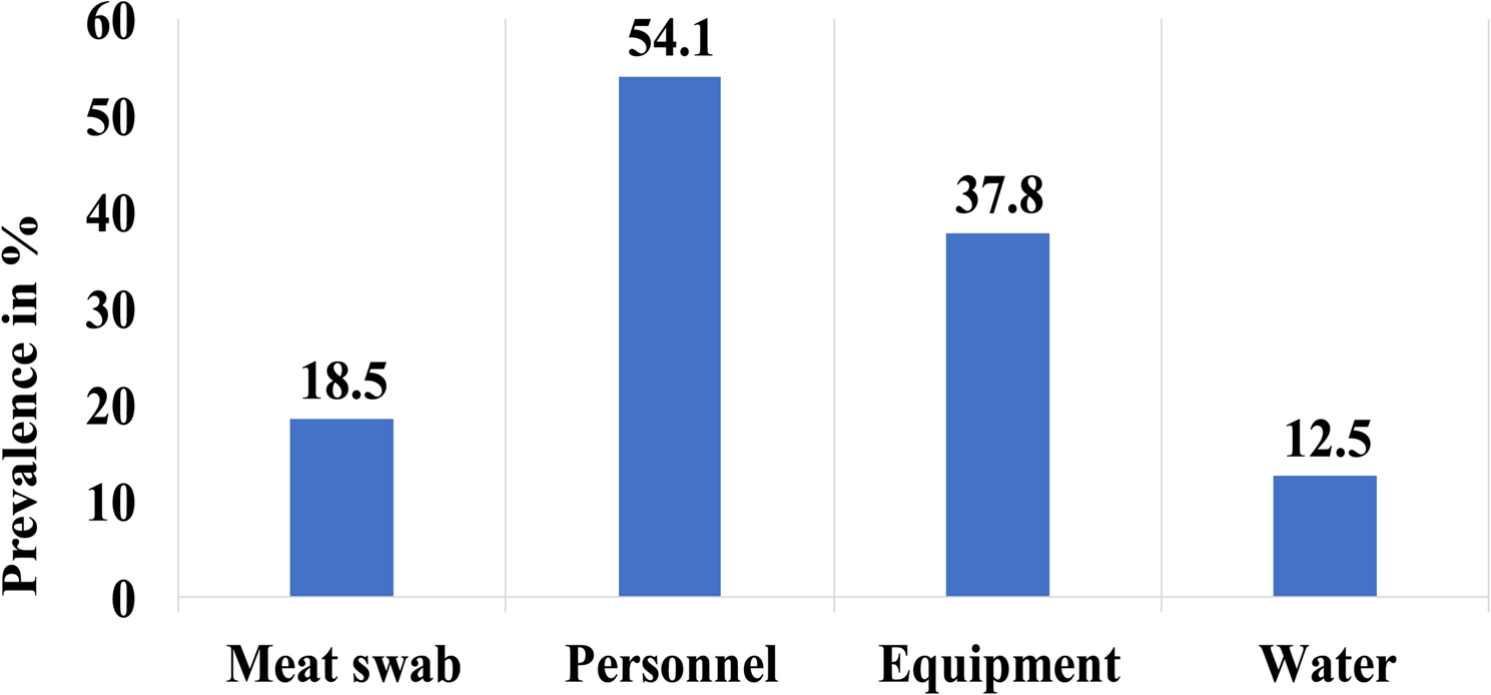
Major categories of samples with their prevalence S. aureus contamination at the export abattoir in Mekelle, Ethiopia

In this study, out of the 400 samples examined, 117 (29.3%) were positive for *Staphylococcus aureus* (*S. aureus*). Meat swabs accounted for 233 of the samples, of which 43 (18.5%; 95% CI: 13.5–23.4) were positive. Within the abattoir personnel samples, *S. aureus* was isolated in 54.1% (95% CI: 40.3–67.4); while equipment samples showed a 37.8% (95% CI: 37.6–57.3) prevalence (Fig. 2).

The prevalence of *S. aureus* differed significantly by type of sample (χ² = 82.9; df = 7; *p* < 0.001). The highest prevalence was observed in knife swabs (83.3%; 95% CI: 64.2–93.3) and hand swabs from abattoir personnel (79.2%; 95% CI: 59.5–90.8), followed by apron swabs (45.8%; 95% CI: 27.9–64.9). A significant difference in contamination was also observed by sample source, with samples from the cattle slaughterhouse showing a higher prevalence of *S. aureus* (33.7%; 95% CI: 27.9–39.6) compared to those from the shoat slaughterhouse (21.6%; 95% CI: 15.0-28.3) (χ² = 6.606; df = 1; *p* < 0.05). Similarly, the prevalence varied significantly by time of collection, with higher contamination detected during non-fasting periods (35.2%; 95% CI: 28.5–41.8) compared to fasting periods (23.4%; 95% CI: 18.1–27.7) (χ² = 6.720; df = 1; *p* < 0.05). In contrast, no statistically significant difference in *S. aureus* prevalence was observed among animal species (χ² = 1.784; df = 2; *p* > 0.05) (Table 1).

**Table 1:** Prevalence and Chi-square (χ²) analysis of factors associated with *S. aureus* contamination at the export abattoir in Mekelle, Ethiopia

Factor	Category	Examined (*n*)	Positive (*n*, %)	95% CI (%)	χ²	df	*p*-value
Type of Sample	Meat Swabs	233	43 (18.5)	13.5–23.4	82.9	7	< 0.001
	Nasal Swabs	24	7 (29.2)	14.9–49.2			
	Hand Swabs	24	19 (79.2)	59.5–90.8			
	Water	24	3 (12.5)	4.3–31.0			
	Knife Swabs	24	20 (83.3)	64.2–93.3			
	Apron Swabs	24	11 (45.8)	27.9–64.9			
	Splitting Axe	23	8 (34.8)	18.8–55.1			
	Slaughter Line	24	6 (25.0)	12.0-44.9			
Sample Source	CSH	252	85 (33.7)	27.9–39.6	6.606	1	0.010
	SSH	148	32 (21.6)	15.0-28.3			
Time of Collection	NFP	199	70 (35.2)	28.5–41.8	6.720	1	0.010
	FP	201	47 (23.4)	18.1–27.7			
Animal Species	Cattle	159	33 (20.8)	15.2–27.7	1.784	2	0.410
	Goat	50	7 (14.0)	7.0-26.2			
	Sheep	24	3 (12.5)	4.3–31.0			

*CSH* Cattle Slaughter House, *SSH* Shoat Slaughter House, *NFP* Non-fasting Period, *FP* Fasting Period

Univariable and multivariable logistic regression analyses were performed to identify factors associated with *S. aureus* contamination (Table 2). In the univariable analysis, type of sample, sample source, and time of collection were significantly associated with *S. aureus* positivity, whereas animal species showed no significant association and was not retained in the multivariable model.

The multivariable logistic regression model showed that, type of sample remained an independent predictor of *S. aureus* contamination. Compared with slaughter line samples, significantly higher odds of contamination were observed for hand swabs (AOR = 3.61; 95% CI: 1.24–10.47; *p* < 0.05) and knife swabs (AOR = 4.92; 95% CI: 1.71–14.12; *p* < 0.05). Other sample types, including meat swabs, nasal swabs, water samples, apron swabs, and splitting axe swabs, were not significantly associated with contamination after adjustment (*p* > 0.05). Sample source was also significantly associated with *S. aureus* isolation in the adjusted model, with samples from the cattle slaughterhouse showing higher odds of contamination compared to those from the shoat slaughterhouse (AOR = 1.74; 95% CI: 1.07–2.82; *p* < 0.05). Similarly, samples collected during non-fasting periods had significantly higher odds of *S. aureus* contamination than those collected during fasting periods (AOR = 1.69; 95% CI: 1.06–2.70; *p* < 0.05) (Table 2).

**Table 2:** Univariable and multivariable logistic regression analysis of factors associated with *S. aureus* contamination at the export abattoir in Mekelle, Ethiopia

Factor	Category	Examined (*n*)	Positive (*n*, %)	COR (95% CI)	*p*-value	AOR (95% CI)	*p*-value
Type of sample	Meat Swabs	233	43 (18.5)	0.68 (0.25–1.81)	0.444	0.72 (0.26–1.98)	0.530
	Nasal Swabs	24	7 (29.2)	1.24 (0.35–4.43)	0.759	1.18 (0.32–4.28)	0.800
	Hand Swabs	24	19 (79.2)	4.00 (2.79–9.95)	0.001	3.61 (1.24–10.47)	0.018
	Water	24	3 (12.5)	0.43 (0.09–1.96)	0.271	0.48 (0.10–2.28)	0.360
	Knife Swabs	24	20 (83.3)	6.00 (3.01–11.34)	0.001	4.92 (1.71–14.12)	0.003
	Apron Swabs	24	11 (45.8)	2.54 (0.75–8.63)	0.137	2.21 (0.62–7.89)	0.220
	Splitting Axe	23	8 (34.8)	1.60 (0.45–5.65)	0.467	1.43 (0.39–5.26)	0.590
	Slaughter Line	24	6 (25.0)	1*	-	1*	-
Sample Source	CSH	252	85 (33.7)	1.85 (1.15–2.95)	0.010	1.74 (1.07–2.82)	0.024
	SSH	148	32 (21.6)	1*	-	1*	-
Time of Collection	NFP	199	70 (35.2)	1.78 (1.15–2.75)	0.010	1.69 (1.06–2.70)	0.027
	FP	201	47 (23.4)	1*	-	1*	-
Animal Species	Cattle	159	33 (20.8)	1.83 (0.52–6.52)	0.350	-	-
	Goat	50	7 (14.0)	1.14 (0.27–4.86)	0.854	-	-
	Sheep	24	3 (12.5)	1*	-	-	-

*CSH* Cattle Slaughter House, *SSH* Shoat Slaughter House, *NFP* Non-fasting Periods, *FP* Fasting Periods

1*: Reference

### Antimicrobial susceptibility testing

Twenty representative *S. aureus* isolates were tested for susceptibility to nine antimicrobial agents from nine different classes. No resistance was observed to ciprofloxacin, chloramphenicol, or sulfamethoxazole-trimethoprim. In contrast, penicillin-G showed the highest resistance rate (90%). An extent of 25% of the isolates also showed resistance to cefoxitin, which are methicillin-resistant *S. aureus* (MRSA). The antimicrobial susceptibility test results for the isolated *S. aureus* are summarized in Table 3.

**Table 3:** Susceptibility patterns of *S. aureus* (*n* = 20) isolated from the export abattoir in Mekelle, Ethiopia

Antimicrobial	Disc potency (ug)	Susceptible *n* (%)	Intermediate *n* (%)	Resistant *n* (%)
Doxycycline	30	11 (55)	5 (25)	4 (20)
Penicillin G	10 units	2 (10)	-	18 (90)
Erythromycin	15	14 (70)	2 (10)	4 (20)
Trimethoprim-sulfamethoxazole	25	20 (100)	-	-
Chloramphenicol	30	20 (100)	-	-
Ciprofloxacin	5	20 (100)	-	-
Gentamicin	10	18 (90)	-	2 (10)
Cefoxitin	30	15 (75)	-	5 (25)
Vancomycin (MIC)	-	17 (85)	1 (5)	2 (10)

With respect to the resistance to more than three class classes of drugs, the *S. aureus* isolates demonstrated Multidrug resistance (MDR). Among the resistant isolates, 4 out of 20 (20%) were identified as multidrug resistant. On the other hand, seven (7) isolates (35%) were resistant to two classes of antimicrobials, while eight (8) isolates (40%) were resistant to only one class of antimicrobials (Table 4).

**Table 4:** Class-based resistance patterns of *S. aureus* (*n* = 20) from the export abattoir in Mekelle, Ethiopia

No. of antimicrobial classes	Antimicrobial classes involved	Resistant antimicrobials	No. of isolates (%)
Resistance in 1 class	Penicillins	PG only	8 (40%)
Resistance in 2 classes	Penicillins + Cephamycins	PG, CFX	2 (10%)
	Penicillins + Macrolides	PG, E	2 (10%)
	Penicillins + Tetracyclines	PG, DO	1 (5%)
	Penicillins + Aminoglycosides	PG, GEN	2 (10%)
Resistance in 3 classes	Penicillins + Cephamycins + Macrolides	PG, CFX, E	1 (5%)
	Penicillins + Tetracyclines + Glycopeptides	PG, DO, VA	1 (5%)
	Cephamycins + Tetracyclines + Macrolides	CFX, DO, E	1 (5%)
	Penicillins + Cephamycins + Tetracyclines + Glycopeptides	PG, CFX, DO, VA	1 (5%)
Total MDR			4 (20%)

MDR was defined as non-susceptibility to ≥ 1 agent in ≥ 3 antimicrobial classes

*PG* Penicillin-G, *DO* Doxycycline, *GEN* Gentamicin, *E* Erythromycin, *VA* Vancomycin, *CFX* Cefoxitin

## Discussion

*Staphylococcus aureus* (*S. aureus*) is an opportunistic pathogen in animals and humans that is known for acquiring AMR [38, 39]. It causes skin infection, mastitis and severe diseases in farm animals and wound infections in humans [40, 41]. It is also well known for causing food poisoning via enterotoxins [10, 13]. In this study, the organism was detected across meat, personnel, water, and equipment surfaces within the abattoir, indicating multiple potential points of contamination. The notably higher recovery from personnel and equipment reveals the critical role of hygiene practices and handling procedures in pathogen transmission within slaughter facilities [42, 43].

The prevalence of *S. aureus* of this study (29.3%) was comparable to a 30% prevalence of *S. aureus* in hanging and minced meat in Adigrat, Tigray, Ethiopia [44]. However, it was lower than 68% in Thailand [45], 54.4% in ready-to-eat meat in Bahrdar, Ethiopia [46], 36.4% in beef carcass swabs and equipment in Asella, Ethiopia [47], and 33% in sheep and goat carcasses in Addis Ababa, Ethiopia [48]. These differences may be attributed to variations in sample types and abattoir hygiene, as poor hygiene promotes microbial contamination. In contrast, this study’s overall prevalence was higher than 15% reported from municipal abattoirs and butcher shops in Addis Ababa, Ethiopia [9], 13.2% in Addis Ababa, Ethiopia [49], 23.4% in Asella, Ethiopia [50], 12.1% from minced meat in Jimma, Ethiopia [51], 10.6% from beef in the Netherlands [52], 20.5% from beef in the USA [53], and 21.2% from abattoirs and butcher shops in Mekelle, Ethiopia [1]. These differences in prevalence may reflect variations in food handling practices, environmental hygiene, and awareness of microbial contamination. Notably, high *S. aureus* contamination is often linked to poor personal hygiene during food handling and processing [54].

In this study, sample type was strongly associated with *S. aureus* contamination (χ² = 82.9; *p* < 0.05), with particularly high odds observed for knife swabs (AOR = 4.92; 95% CI: 1.71–14.12; *p* < 0.05) and hand swabs (AOR = 3.61; 95% CI: 1.24–10.47; *p* < 0.05) compared to slaughter line swabs. These results reveal abattoir personnel and equipment as major reservoirs of contamination. Comparable findings have been reported in Ethiopia, where Adugna et al.. documented significantly higher odds of *S. aureus* from knives and butcher hands (AOR = 2.8 and 2.4, respectively) compared to carcass samples [9], and Gizaw et al.. similarly observed elevated prevalence in knife and hand swabs along dairy and beef production lines [55].

This study’s knife swab odds of contamination are higher than values previously reported locally, suggesting either poorer tool sanitation or higher workload pressure in the studied facility. International studies also support this pattern: in Hong Kong, Ho et al.. found hand carriage of *S. aureus* to be a strong risk factor for cross-contamination among food handlers (OR = 5.66) [56], while another abattoir study in Thailand reported smaller but significant associations between inadequate knife sanitization and contamination (OR ≈ 1.5) [57]. Likewise, Wardhana et al.. in Indonesia showed that poor hygiene practices increased contamination risk (OR ≈ 2.7) [58]. To reduce cross-contamination, mandatory hand-washing stations with disinfectants, routine knife sterilization between carcasses, and the use of color-coded knives for specific processing tasks are important, alongside regular hygiene training and supervision [59].

Temporal variation was also significant in our study, with samples collected during non-fasting periods showing higher contamination (AOR = 1.69; 95% CI: 1.06–2.70; *p* < 0.05). While few Ethiopian studies have compared fasting vs. non-fasting periods directly, this finding aligns with the broader evidence that busy slaughter seasons, higher throughput with increased worker turnover along the slaughter line, reduced intervals for hand-washing and rushed handling increase contamination rates [60]. Such operational pressure likely increased opportunities for cross-contamination, consistent with evidence linking high slaughter throughput and rushed handling to elevated microbial contamination [61, 62].

Species of animal did not significantly predict contamination (*p* > 0.05), consistent with studies indicating that cross-contamination is driven more by hygiene and environment than by inherent species factors [55, 58]. Taken together, these results of high contamination in hands of personnel, knives, aprons, slaughter-line surfaces, animals coming contaminated with *S. aureus* associated with poor hygiene can act as primary and secondary routes that disseminate *S. aureus* along the processing chain. This echo both national and international evidence that poor personal hygiene and inadequate sanitation are central drivers of *S. aureus* contamination in the meat chain [42, 62].

The high prevalence of *S. aureus* among abattoir workers (54.1%) and on equipment surfaces (37.8%) reveals how human, animal, and environmental pathways intersect within the slaughterhouse system. This overlap emphasizes the need for a One Health approach, where surveillance and interventions are coordinated across public health, veterinary services, and environmental hygiene teams. Strengthening joint monitoring, improving worker hygiene practices such as frequent hand-washing with disinfectant, and enforcing equipment sanitation are all essential elements of intersectoral control that directly align with the One Health framework [43].

In the present study, the isolates demonstrated varying susceptibilities to the tested antimicrobial agents. The antimicrobial sensitivity findings of this study were in agreement with those of a study in Adama, Ethiopia [63], which reported high susceptibility of *S. aureus* to chloramphenicol (100%). The effectiveness of chloramphenicol (100%), ciprofloxacin (100%), and sulfamethoxazole-trimethoprim (100%) against *S. aureus* was also consistent with that reported in a study from Ethiopia [48]. Similarly, the 100% sensitivity of *S. aureus* to chloramphenicol and sulfamethoxazole-trimethoprim was also similar to that reported in another study in Ethiopia [47]. This full susceptibility of *S. aureus* could be basically due to the reduced use pattern of these antibiotics in Ethiopia and the study area [64, 65]. However, this sensitivity was greater than that reported in a study conducted in Assela, Ethiopia [50], where 68.2%, 81.8% and 86.4% of the isolates were susceptible to chloramphenicol, ciprofloxacin and sulfamethoxazole-trimethoprim, respectively.

The markedly high resistance to penicillin-G observed in this study (90%) is basically due to the ability of *S. aureus* to produce β-lactamase, predominantly mediated by the *blaZ* gene [66]. Such high resistance to penicillin-G among *S. aureus* isolates has been repeatedly documented in Ethiopia; for example, Gizaw et al.. found 95% penicillin resistance in isolates from dairy and meat supply chains, and other studies in milk, meat, and abattoir settings reported similar rates (87–95%) [67–69]. On the other hand, the 90% resistance of *S. aureus* isolates to penicillin-G was greater than 67.9% in Hawassa, Ethiopia [70], 86.9% resistance pattern in another Ethiopian study [48], 71.6% in South Africa [71], and 69.1% in Italy [45]. This high resistance to penicillin G in the study area could be largely driven by the widespread and often unregulated use of older β-lactams in both veterinary and human sectors [72, 73], though the resistance level to penicillin-G resistance in this study was lower than 100% resistance in Gujarat [74].

In this study, 25% of *S. aureus* isolates were phenotypically cefoxitin-resistant (used as a surrogate for MRSA), a prevalence higher than pooled global estimates for meat and meat products (2.75–5.02%) [22], but lower than reports from other Ethiopian meat-chain studies (41.3–55.8%) [5, 67], indicating variability in hygiene practices and antimicrobial exposure across facilities. Vancomycin resistance was detected in 10% (2/20) of isolates, which is concerning given vancomycin’s role as a last-line therapy for severe MRSA infections [75]. Comparable Ethiopian studies have reported pooled vancomycin-resistant *S. aureus* (VRSA) prevalence of 14.5% among clinical isolates [76] and up to 65.1% in abattoir and dairy settings [5]. The detection of MRSA and VRSA on carcasses, equipment, and personnel hands reveals significant food-safety and occupational-health risks, emphasizing the need for strengthened hygiene measures, antimicrobial stewardship, and integrated One Health surveillance to curb the spread of resistant strains [77, 78].

Four isolates (4/20, 20%) showed Multidrug resistance (MDR) to at least one agent in three or more antimicrobial classes. This figure is higher than a report by Tibebu et al.. from Bishoftu, Oromia, Ethiopia with overall MDR 10.4% [79]. However, the MDR figure of this study was lower than that of another studies conducted in Ethiopia, like 73.9% [67], 53.3% [80], 34.4% [48], 62.8% [70], 98% [46] and 100% [49]. The comparatively lower MDR prevalence observed in this study may be partly attributed to differences in study setting, sample size, antimicrobial agents tested, and antimicrobial use practices. Unlike studies conducted in clinical settings or intensive production systems, this investigation was based on an export abattoir environment, where the animals regularly undergone pre-slaughter selection and withdrawal periods that reduce antimicrobial pressure. In addition, only a subset of isolates underwent antimicrobial susceptibility testing, which may have limited the detection of rarer MDR phenotypes.

### Limitations of the study

This study has some limitations that should be acknowledged. First, molecular characterization and analysis of specific virulence factors were not performed due to resource constraints. Specifically, methicillin resistance was assessed phenotypically using cefoxitin disk diffusion without confirmation of the *mecA* or *mecC* genes, and no molecular typing methods such as *spa* typing or multilocus sequence typing (MLST) were conducted. This limits the molecular confirmation and ability to compare isolates at the genetic level. Second, the small antimicrobial susceptibility test sample size due to resource constraints may limit the AMR and MDR generalizability of the *S. aureus* isolates recovered in this study. Third, the use of convenience sampling for personnel and water samples, may also introduced selection bias. Fourth, the study was confined to a single export abattoir in northern Ethiopia and the findings may not represent other regions or types of slaughter facilities. Despite these limitations, the study provides baseline data on *S. aureus* contamination and antimicrobial susceptibility in an export abattoir setting in northern Ethiopia and reveals critical hygiene and food-safety gaps. Future studies should aim to include larger antimicrobial susceptibility testing samples and employ molecular typing methods to better characterize the genetic diversity and transmission dynamics of isolates.

## Conclusion

In this study, *Staphylococcus aureus* (*S. aureus*) was detected in meat, abattoir personnel, equipment, and water, with an overall prevalence of 29.3% (117/400). The findings highlighted poor hygiene practices that increase the risk of meat borne infections. Contamination was observed on hands, utensils (such as knives), and water, indicating inadequate hygiene during processing and a failure to sterilize equipment. This raises concerns about raw meat acting as a reservoir for antibiotic-resistant *S. aureus*, which poses a serious public health threat. Some isolates exhibited Multidrug resistance (MDR) to at least one agent in at least three class of antimicrobials. Improving hygienic practices at abattoirs is vital for safeguarding public health against staphylococcal food poisoning and the transmission of multidrug-resistant strains. In conclusion, the responsible use of antibiotics in both the human healthcare and veterinary sectors through the One Health approach is essential to curb the development of antimicrobial resistance.

## Supplementary Information

Supplementary Material 1.

## Data Availability

The data used and/or analyzed during the study are available from the corresponding author upon reasonable request.

## Abbreviations

AMR: Antimicrobial Resistance
AOR: Adjusted Odds Ratio
MDR: Multidrug Resistance
MIC: Minimum Inhibitory Concentration
MRSA: Methicillin-resistant S. aureus
S. aureus: *Staphylococcus aureus*
VRSA: Vancomycin-resistant S. aureus
