# Supplementary material for: Prevalence and antimicrobial susceptibility profile of Staphylococcus aureus from meat, abattoir workers, equipment and water samples at Abergelle International Livestock Development PLC, Mekelle, Northern Ethiopia
Source: BMC Microbiol. 2026 Jan 15;26:171. doi: 10.1186/s12866-026-04709-1 (PMC12947405; doi:10.1186/s12866-026-04709-1)
Supplement: Supplementary file 1 — Supplementary Material 1. [file 12866_2026_4709_MOESM1_ESM.docx]

Supplementary Table 1. CLSI M100 30^th^ edition interpretive criteria used for antimicrobial susceptibility testing of Staphylococcus aureus

| **Antimicrobial agent** | **Disc potency** | **Susceptible** | **Intermediate** | **Resistant** |
| --- | --- | --- | --- | --- |
| Doxycycline | 30 µg | ≥ 16 mm | 13–15 mm | ≤ 12 mm |
| Penicillin G | 10 units | ≥ 29 mm | - | ≤ 28 mm |
| Erythromycin | 15 µg | ≥ 23 mm | 14–22 mm | ≤ 13 mm |
| Trimethoprim-sulfamethoxazole | 25 µg | ≥ 16 mm | - | ≤ 15 mm |
| Chloramphenicol | 30 µg | ≥ 18 mm | 13–17 mm | ≤ 12 mm |
| Ciprofloxacin | 5 µg | ≥ 21 mm | 16–20 mm | ≤ 15 mm |
| Gentamicin | 10 µg | ≥ 15 mm | 13–14 mm | ≤ 12 mm |
| Cefoxitin | 30 µg | ≥ 22 mm | - | ≤ 21 mm |
| Vancomycin | MIC (µg/mL) | ≤ 2 | 4–8 | ≥ 16 |
